# Egg Load Decreases Mobility and Increases Predation Risk in Female Black-Horned Tree Crickets (*Oecanthus nigricornis*)

**DOI:** 10.1371/journal.pone.0110298

**Published:** 2014-10-15

**Authors:** Kyla Ercit, Andrew Martinez-Novoa, Darryl T. Gwynne

**Affiliations:** 1 Ecology and Evolutionary Biology, University of Toronto at Mississauga, Mississauga, Ontario, Canada; 2 Department of Biology, University of Toronto at Mississauga, Mississauga, Ontario, Canada; CNRS, France

## Abstract

Female-biased predation is an uncommon phenomenon in nature since males of many species take on riskier behaviours to gain more mates. Several species of sphecid wasps have been observed taking more female than male prey, and it is not fully understood why. The solitary sphecid *Isodontia mexicana* catches more adult female tree cricket (*Oecanthus nigricornis*) prey. Previous work has shown that, although female tree crickets are larger and thus likely to be more valuable as prey than males, body size alone cannot fully explain why wasps take more females. We tested the hypothesis that wasps catch adult female tree crickets more often because bearing eggs impedes a female’s ability to escape predation. We compared female survivors to prey of *I. mexicana*, and found that females carrying more eggs were significantly more likely to be caught by wasps, regardless of their body size and jumping leg mass. We also conducted laboratory experiments where females’ jumping responses to a simulated attack were measured and compared to her egg load and morphology. We found a significant negative relationship between egg load and jumping ability, and a positive relationship between body size and jumping ability. These findings support the hypothesis that ovarian eggs are a physical handicap that contributes to female-biased predation in this system. Predation on the most fecund females may have ecological-evolutionary consequences such as collapse of prey populations or selection for alternate life history strategies and behaviours.

## Introduction

Sex- biased predation is common in nature, and can have significant evolutionary consequences for prey. For example, male-biased predation can lead to rapid evolution of new phenotypes and life-history strategies (e.g. [1]). While much is known about the causes and consequences of male-biased predation [2], [3], female-biased predation is comparatively uncommon and requires further understanding, especially since female-biased mortality can cause unstable population dynamics which potentially leads to population collapse [4]. Female-biased predation is evolutionarily significant because it can lead to changes in prey behaviour and life history strategies. In populations at high risk of predation, females can prefer less conspicuous mates [5], or become less receptive to courtship [6], and this, in turn, can lead to the evolution of alternative mating strategies by males [7].

Broadly, the mechanisms of female-biased predation can be classified as either predator-mediated (that predators have a preference for or an improved ability to catch females) or prey-mediated (morphology or life-history traits of females put them at greater risk of predation), but many instances of female-biased predation will likely be influenced by both mechanisms. Life-history traits that can lead to increased predation on females include female-biased sex size dimorphism [6], [8]; risky mating behaviour such as mate-search [9], intrasexual competition [10] or copulation [11]–[13]; or risks associated with rearing young, such as trading-off vigilance for increased foraging [14], [15], and guarding eggs or young [16]. The physical stress and morphological changes associated with carrying young or eggs may also lead to increased female mortality. Gravid or ovigerous females are often reported to suffer higher predation [17]–[21]. This increased mortality can sometimes be attributed to the physical handicap of females carrying and developing eggs [17], [22], [23]. Furthermore, in species where females transfer eggs to a conspecific, it is often the egg-bearing individual that suffers from increased predation (e.g. Pipefish *Nerophis ophidion*: [24], Golden egg bug *Phyllomorpha laciniata*: [25]).

Although female-biased predation occurs infrequently among insects in general [4], it occurs commonly among arthropods hunted by sphecid and crabronid wasps as fresh (paralysed) provisions for wasps’ larvae [11], [26]–[31]. *Isodontia mexicana*, a sphecid, has been observed provisioning its nest with more female than male tree crickets (*Oecanthus nigricornis* and *O. quadripunctatus*) [32], [33]. The *I. mexicana* and tree cricket system is an ideal one for examining the causes of sex-biased predation, since these wasps will nest in artificial trap nests [38], allowing many of their intact prey to be sampled. *I. mexicana* prefer larger prey, and although size accounts in part for the female bias, when body size is controlled statistically, female crickets are still more likely to be taken than males [34]. It is possible that sex-specific traits of female crickets, in particular ovarian eggs, make them more vulnerable to wasp predation. A common response to predator stimulus among orthopterans is jumping to evade the predator [35], [36]. In jumping orthopterans, jump performance depends on both leg muscle mass and body mass [37], and females with more eggs are expected to be more constrained when jumping to escape a predator. In this paper, we test this hypothesis, predicting first that female crickets caught by wasps have more ovarian eggs than survivors, and second, that more eggs decrease a female’s mobility by restricting jumping distances.

## Methods

**Figure 1:**
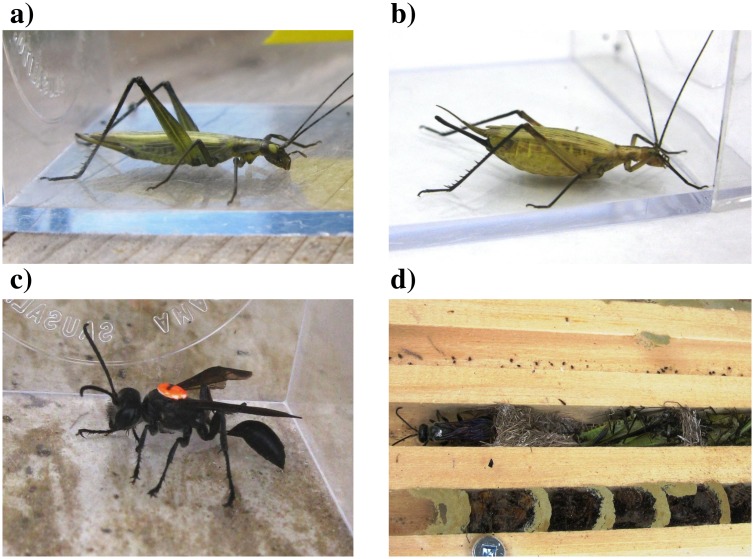
Adult female tree crickets *Oecanthus nigricornis* with *a)* few ovarian eggs and *b)* an abdomen full of ovarian eggs; *c)* Predaceous wasp *Isodontia mexicana* (marked with a bee tag); and *d) I. mexicana* (marked with white paint) in a nest bore she is provisioning with paralysed *O. nigricornis* crickets. Below her is a nest provisioned with spiders by *Trypoxylon lactitarse*. Photos by K. Ercit.

Black-horned tree crickets (*Oecanthus nigricornis*) are common and widespread orthopterans in meadows of North America [39], [40]. They are univoltine in Canada, and adults are present between July and September. Females are polyandrous and receive a nutritionally valuable nuptial gift during courtship [41] from metanotal gland secretions consumed during and after courtship [42]. A mated female *O. nigricornis* chews a hole into the stem of a suitable plant (usually raspberry, goldenrod, or sumac), and digs a chamber into the pith using her ovipositor, into which she deposits an egg [42]. It can take ten to thirty minutes to lay a single egg in this fashion, and a female may lay about a dozen eggs in a single session of oviposition [42]. Mated females may be restricted in unburdening themselves of eggs quickly and they may hold several dozen mature ovarian eggs [42]. Egg-laden female *Oecanthus* often show visibly distended abdomens in nature [43] (Figure 1a and b). *Oecanthus* crickets are often preyed upon by *I. mexicana* [44], a solitary sphecid wasp that paralyzes and provision their nests with crickets and small katydids [45] (Figure 1c and d).

Between 2009 and 2012, we placed out artificial trap-nests each season in tree cricket habitat at the University of Toronto Koffler Scientific Reserve (KSR) in King City, ON (44°03′N, 79°54′W). Trap-nests were blocks of wood with drilled bores, and topped with acrylic lids to allow viewing of contents [46]. Thirty-five blocks were stacked in a box on a platform about 1 m off the ground, and covered with a shingled roof. Every year, from the beginning of the *I. mexicana* nesting season (mid- to late- July), recently-provisioned prey were sampled approximately weekly from trap-nests. At these times, surviving tree crickets were also sampled via haphazard net sweeps in the habitat surrounding the trap nests. All adult female samples were sacrificed and preserved in 95% ethanol until they could be dissected. See [34] for a more detailed description of the trap-nest setup and sampling. In 2011, there were too few adult female crickets found in *I. mexicana* nests (8 crickets, likely caught by only 2 different wasps) to perform any meaningful comparison, so these data were omitted from the analysis.

To test whether females with more eggs are less mobile than their less fecund sisters, we reared 29 female *O. nigricornis* to adulthood in the lab and tested their jumping performance. Crickets were caught as 4^th^ or 5^th^ instar nymphs from a wild population on the University of Toronto at Mississauga campus in the summer of 2013. Captive crickets were housed in separate 10 cm diameter Plexiglas cylinders and given food (pollen, cat food, and occasionally apple) and water *ad libidum*, as well as 10 cm sections of raspberry stem as oviposition substrate. Jumping trials commenced two weeks after all crickets had reached maturity. For each trial, an individual cricket was placed in the centre of a 48 cm×62 cm paper-lined arena. To prevent contact pheromones from previous subjects affecting behaviour, paper was changed between trials. Each cricket was allowed 15 minutes to habituate to the arena, and then a predator attack was simulated by brushing her hind leg with a paintbrush. The technician who simulated predation was blind to the hypothesis of the experiment. Female jumps were recorded by a camera (Casio EX-S7) on a tripod positioned downwards towards the inside of the arena. The tracking software Kinovea (version 0.8.15) was used to replay the recordings on a computer and measure the cricket’s jump distance. Each female was tested twice, and the longer of the two jumps was used in statistical analysis. Following the trials, crickets were sacrificed and preserved in 95% ethanol until they could be dissected and measured.

The ovaries of all adult female *O. nigricornis* from field sampling and laboratory jump trials were dissected and mature eggs were counted to determine egg load. Mature eggs were identified as being smooth, whitish-yellow, with a visible, differentiated cap. Females with internal parasites were removed from further analysis. The hind jumping legs of all females from field sampling and lab trials were removed and dried in a desiccating oven at 50°C for 75 minutes (15 minutes after any change in mass was detectible) and weighed on a Mettler Toledo balance. Dry leg mass was used instead of wet mass to correct for differences in liquid loss or absorption between older and more recently collected crickets due to storage in ethanol. Although body mass of crickets is likely an important variable in predicting jumping performance [37], we did not include it in our analysis because paralysed crickets sampled from wasp nests necessarily have lower mass than survivors due to excreting but not ingesting. Instead we measured pronotum length as a proxy for overall body mass, since the two traits are strongly correlated [34]. Pronotum length was measured using ImageJ software from photographs taken by an AmScope 5 MP digital camera mounted to a Wild Heerbrugg M5A dissecting microscope.

All statistics were computed using R version 3.0.2 [47]. To determine if egg load, leg mass, and body size affect a females’ chance of surviving wasp predation in the wild, we used logistic regression of a generalized linear model. We first tested a model with leg mass, egg number, and pronotum length, pair-wise interactions between morphological variables, sampling year, sampling date as a blocking factor. We removed non-significant terms from the model using the R function “step”, and removed terms with a variance inflation score over 20. To compare day effects across the years, we converted sampling date to relative date: we numbered the first day of each year that adult female survivors and prey were collected as day 1 and the last day as day n. We then divided each day number by n and multiplied it by the days in the longest sampling season (22 days).

To determine the effect of female cricket egg load, leg mass, and body size on jump distance in the lab, we performed a multiple regression. Leg mass was log-transformed to conform to a normal distribution. We started by testing a model of the effect of egg number, leg mass, pronotum length, and all pair-wise interactions on jump distance. We removed non-significant terms from the model using the R function “step”, and removed terms with a variance inflation score over 20.

## Results

**Figure 2:**
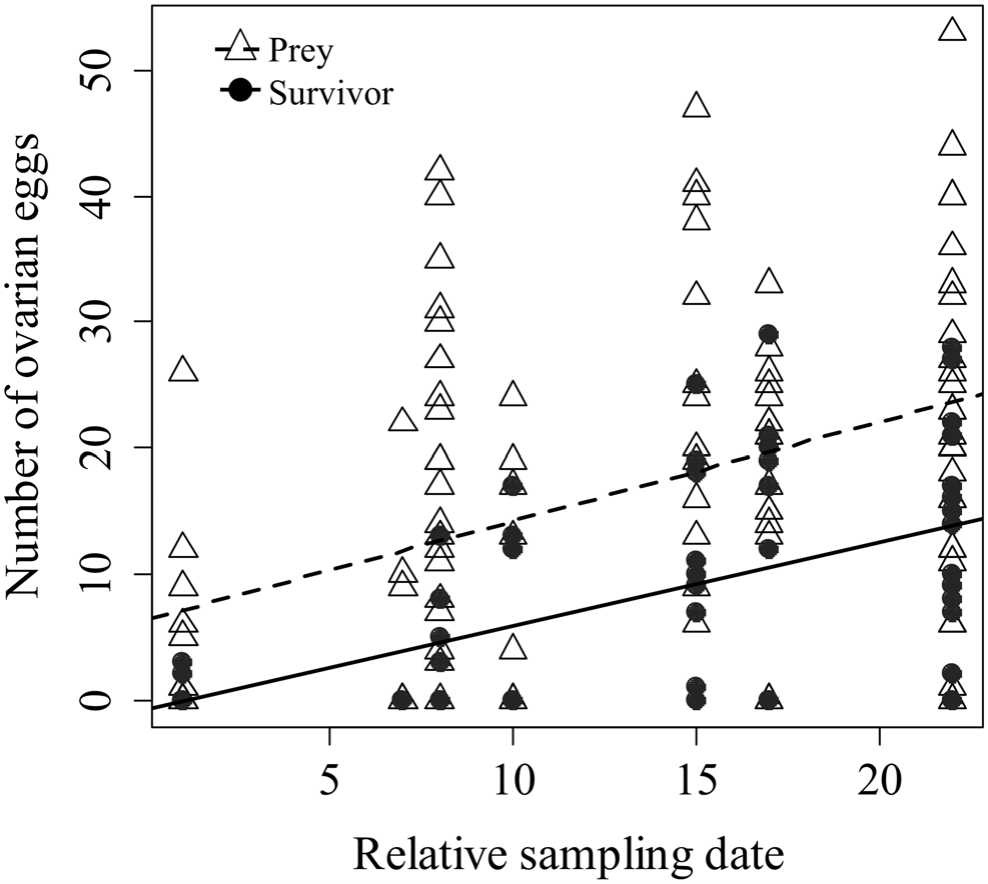
Number of mature ovarian eggs from wild female *Oecanthus nigricornis* that were prey (triangles and dashed line) and survivors (filled circles and solid line) of *Isodontia mexicana*.

The number of eggs in a female tree cricket was significantly negatively related to her chances of surviving wasp predation in the wild (*β* = −0.10, *P*<0.01) (Table 1b). Neither leg mass, body size, year nor any interactions between morphological variables were significantly related to chance of survival. In every sampling year, prey females held more eggs than survivor females (Table 2 and Figure 2). As reported in a previous publication [34], wasps took significantly more female prey than was representative of the cricket population (2009: prey 1∶2.3 (m:f), survivors 1∶1.3; 2010: prey 1∶1.8, survivors 1∶1.3; 2012 prey 1∶2.3, survivors 1∶1.1).

**Table 1:** Results of multiple regressions testing variables of female *Oecanthus nigricornis* that explain *a)* the distance they can jump; and *b)* the chance of surviving predation in the wild by *Isodontia mexicana* wasps.

*a) Lab experiment*			
n = 29	Term	Coefficient	*P*
	Intercept	−19.2	0.17
	Leg mass (mg)	0.88	0.91
	**Egg number**	−0.11	<0.01
	**Pronotum length (mm)**	18.1	0.02
***b) Field sampling***			
**n = 189**	**Term**	**Coefficient**	***P***
	Intercept	1.01	0.73
	**Egg number**	−0.10	<0.01
	Leg mass (mg)	1.29	0.18
	Pronotum length (mm)	−1.19	0.42
	**Relative sampling date**	0.07	0.01

The original regression models contained leg mass, ovarian egg number, pronotum length, all pair-wise interaction terms, and (for the field data) year, and sampling date as a blocking factor. Non-significant interaction terms were removed via model simplification. Bold terms indicate significance at α = 0.05.

**Table 2:** Summary statistics of morphological traits and number of mature eggs in female *Oecanthus nigricornis* reared in the lab as well as those from a wild population that survived and succumbed to predation by wasp *Isodontia mexicana*.

	Median and IQR	Mean ±SEM	
	Egg number	Pronotum length (mm)	Leg mass (mg)
Lab-reared	39 (21–69)	2.30±0.022	1.13±0.042
2009 Survivor	4 (0–11)	2.32±0.019	1.26±0.041
2009 Prey	19 (6–30)	2.34±0.018	1.32±0.031
2010 Survivor	0 (0–10)	2.51±0.024	1.41±0.063
2010 Prey	10 (0–21)	2.50±0.031	1.43±0.056
2012 Survivor	13 (0–17)	2.51±0.022	1.58±0.054
2012 Prey	17 (5–24)	2.52±0.020	1.57±0.042

**Figure 3:**
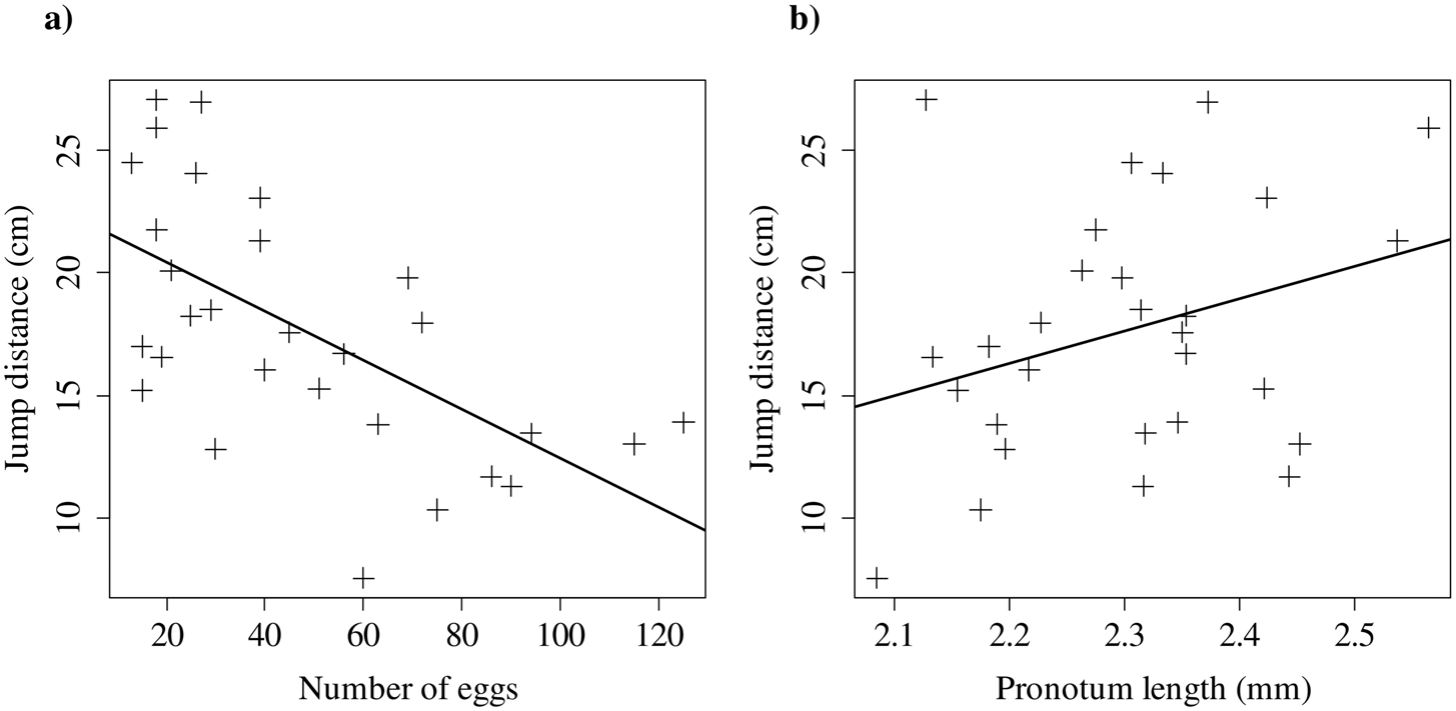
The relationship between *a)* the number of ovarian eggs and *b)* the pronotum length (as a proxy for body mass) and the distance a female *Oecanthus nigricornis* can jump in laboratory conditions.

Lab-reared females had more eggs than those found in the wild, and were smaller in general (Table 2). Multiple regression revealed that egg number and pronotum length significantly affected jump distance: pronotum length affected jump distance positively (*β* = 18.1, *P* = 0.02), and the number of eggs affected jump distance negatively (*β* = −0.11, *P*<0.01) (Figure 3). Neither leg mass nor any interactions between variables had a significant effect on jump distance (Table 1a). Multicollinearity was not a significant problem in either field or laboratory analysis: in the final, simplified models, variance inflation scores of any term did not exceed 3.

## Discussion

Both of our initial predictions were supported: in the wild, the number of mature ovarian eggs had a significant negative relationship with a female cricket’s chances of surviving wasp predation. Also, in laboratory experiments, the number of mature ovarian eggs had a negative effect on jump distance. These findings support the hypothesis that female-biased predation of adult *O. nigricornis* by wasps is influenced by female prey being physically handicapped by ovarian eggs. We cannot rule out the hypothesis that wasps actively prefer ovigerous females, as observed in the jumping spider *Portia labiata* hunting its spitting spider prey, *Scytodes pallida* [48]. It is also possible that wasps take females with more eggs because they are larger and easier for a predator to detect. We included pronotum length in our analyses as proxy of body mass, and we found no effect of pronotum length on risk of capture by wasps, but egg-laden females with distended abdomens [43], may be more detectable.

We note that pronotum length had a positive effect on jump distance in the laboratory experiment, but did not find evidence of this relationship in the wild. Although larger-bodied crickets in the wild may be able to jump farther, these crickets may be more detectable by predators. This discrepancy may also be due to the fact that animals often perform differently in nature versus in laboratory experiments [49]. Our finding that leg mass had no detectable effect on either jump performance or survival in the wild is consistent with results for another jumping orthopteran, the grasshopper *Melanoplus femurrubrum*, where improvement in jumping performance and predation avoidance was not due to differences in leg size [35].

The greater predation risk for egg-laden females may explain why other solitary wasps often take more female prey. Not only would egg-burdened prey take less energy to catch, but there are probably nutritional benefits of large, egg-laden prey because they are nutrient-rich and likely easier to digest [50], and their higher value means fewer foraging trips. Sphecid wasps provision a certain mass of prey per egg rather than a certain number [26], and tend to take as close to the maximum mass of prey that they are able to carry per trip [51]. Energetic benefits likely contribute to the overrepresentation of adult females in the prey of *I. mexicana* and other predatory wasps.

Predation on fecund females is unlikely to result in total selection on prey females for fewer mature eggs as mortality costs are probably countered by sexual or fecundity selection to produce more eggs. Not only are females with more eggs likely to have more surviving offspring, but males also tend to prefer females that appear to bear more eggs [52]. This male preference is especially important in a nuptial gift-giving species like *O. nigricornis* because females that mate more receive more gifts, which can further increase their reproductive output [41]. When multiple mating is beneficial to females, sexual selection may occasionally result in female ornaments that accentuate fecundity, as seen in dance flies *Rhamphomyia longicauda* [53]. Even though these ornaments carry a mortality cost [54], they persist, likely because males are attracted to larger females.

A bias in predation on fecund females by sphecids may hold potentially important consequences for prey populations. In addition to greatly reducing local prey populations [55] and creating unstable population dynamics [4], hunting the most fecund females likely further limits tree cricket population growth. This potentially strong viability selection on females may result in selection for alternative behaviours and life history strategies in females such as delayed reproduction [19], or reduced movement and increased crypsis [56].

## Supporting Information

Data S1 Raw data from the collection of *Oecanthus nigricornis* as survivors and prey of the predaceous wasp *Isodontia mexicana* at Koffler Scientific Reserve, as well as results of laboratory experiments of *O. nigricornis* jump performance. Includes the date of collection, survival status, ovarian egg number, and morphological measurements for field samples; and ovarian egg number, morphological measurements, and results of laboratory jumping trials.
(XLSX)
